# Temperature dependence of nitrification in a membrane-aerated biofilm reactor

**DOI:** 10.3389/fmicb.2023.1114647

**Published:** 2023-04-24

**Authors:** András Németh, Jude Ainsworth, Harish Ravishankar, Piet N. L. Lens, Barry Heffernan

**Affiliations:** ^1^OxyMem Ltd., Athlone, Ireland; ^2^Department of Microbiology, University of Galway, Galway, Ireland

**Keywords:** membrane-aerated biofilm, nitrification rate, wastewater treatment, mass transfer, community composition, temperature dependence

## Abstract

The membrane-aerated biofilm reactor (MABR) is a novel method for the biological treatment of wastewaters and has been successfully applied for nitrification. To improve the design and adaptation of MABR processes for colder climates and varying temperatures, the temperature dependence of a counter-diffusional biofilm’s nitrification performance was investigated. A lab-scale MABR system with silicone hollow fibre membranes was operated at various temperatures between 8 and 30°C, and batch tests were performed to determine the ammonia oxidation kinetics. Biofilm samples were taken at 8 and 24°C and analysed with 16S rRNA sequencing to monitor changes in the microbial community composition, and a mathematical model was used to study the temperature dependence of mass transfer. A high nitrification rate (3.08 g N m^–2^ d^–1^) was achieved at 8°C, and temperature dependence was found to be low (θ = 1.024–1.026) compared to suspended growth processes. Changes in the community composition were moderate, *Nitrospira defluvii* remaining the most dominant species. Mass transfer limitations were shown to be largely responsible for the observed trends, consistent with other biofilm processes. The results show that the MABR is a promising technology for low temperature nitrification, and appropriate management of the mass transfer resistance can optimise the process for both low and high temperature operation.

## 1. Introduction

Many wastewater treatment plants around the world are experiencing increased loads and more stringent effluent requirements, often coupled with ageing infrastructure (Steve and Matthews, 2018; European Union, 2019; ICPDR, 2021). Removal of ammonia, which most often is realised *via* biological nitrification, can become the bottleneck in treatment efficiency, especially in temperate and colder climates where the water temperature in winter can drop below 12°C. Biofilm processes have the potential to intensify nitrification by providing increased solids retention times for slow growing autotrophic organisms, like nitrifiers.

It is well established that nitrification rates decrease concomitantly with decreasing temperature, for example, Knowles et al. (1965) studied nitrifying cultures from the Thames river estuary and found that both *Nitrosomonas* and *Nitrobacter* activity were significantly influenced by temperature in the range of 8–23°C, the growth rate of the former showing a 9.5% increase with each °C of rise in temperature. *Nitrobacter* were less affected and showed about 50% higher growth rate on average compared to *Nitrosomonas*. Antoniou et al. (1990) also investigated the impact of pH and temperature on the effective growth rate of nitrifiers. A positive correlation of growth rate and temperature was shown in the range of 15–25°C at pH values ranging from 6.8 to 8.2. In another study, Characklis and Gujer (1979) pointed out that while the temperature dependency of microbial reactions can be described with an Arrhenius-type correlation, this is valid only for single reactions or ones in which the first step is rate-limiting. The influence of mass transfer was also discussed in relation to bacterial aggregates in wastewater treatment processes (flocs and biofilms), acknowledging that the effect of temperature in such cases is more difficult to analyse.

Biofilms are known to behave as reaction-diffusion systems, where the influence of mass transfer is more significant than in suspended growth cultures, often limiting the overall reaction rate. It is necessary to consider the temperature influence on mass transfer besides its effect on the intrinsic reaction rate. For example, Zhu and Chen (2002) found that in aerated biofilters temperature had a more significant impact on the nitrification rate under ammonium limiting conditions than under oxygen limiting conditions. According to their sensitivity analysis, diffusion contributed more to the temperature effect than the nitrifier growth rate. In another study conducted on a biofilter, Delatolla et al. (2009) observed that the temperature effect depended on the length of exposure to cold temperature, which suggests the influence of shifts in the microbial community composition or activity in addition to the effects of mass transfer. Hoang et al. (2014) observed a kinetic threshold at 5°C in a nitrifying moving bed biofilm reactor (MBBR) when operating for an extended period of time, but found that nitrification could be realised even at 1°C. Ashkanani et al. (2019) investigated the impact of temperature on nitrifying MBBR performance with real secondary effluent, confirming the influence of exposure time to cold temperatures. They also found that carriers showing more clogging, and therefore an increased mass transfer resistance, were less affected by temperature.

Membrane-aerated biofilm reactors (MABRs) rely on a gas-permeable membrane as the substratum to deliver oxygen to the biofilm, while the electron donors (organic carbon or ammonia) diffuse into the biofilm from the bulk liquid. The resulting counter-diffusional biofilm creates a favourable environment for the growth of nitrifiers at the membrane-biofilm interface, where the dissolved oxygen concentration is high, and the concentration of organic substrates is low. The nitrification rate of MABR systems using synthetic wastewater and secondary effluents was studied by various researchers (Brindle and Stephenson, 1996; Brindle et al., 1998; Yamagiwa et al., 1998; Suzuki et al., 2000; Hibiya et al., 2003; Semmens et al., 2003; Terada et al., 2003; Walter et al., 2005; Downing and Nerenberg, 2008; Hwang et al., 2009; Lackner et al., 2010; Castrillo et al., 2019). A wide range of nitrification rates were reported in these studies, 1.1–9.26 g N m^–2^ d^–1^ and loading conditions and bulk liquid ammonia concentration have strongly influenced the results, which is characteristic of the mass transfer limitations in biofilm processes. In addition, factors such as the type of medium; synthetic or real secondary effluent and temperature, ranging from 20 to 30°C, probably contributed to this variability. The volumetric ammonium removal rates were reaching up to 1 kg N m^–3^ d^–1^, but they varied significantly depending on the packing density and operating conditions of the specific reactors.

Previous studies have investigated the effects of oxygen pressure in the lumen, COD:N ratio of the feed, biofilm thickness and mixing conditions. However, no specific studies on the effect of temperature in MABRs have been conducted to date. A better understanding of temperature dependence in nitrifying membrane-aerated biofilms is necessary to help technology providers, designers, and operators in maximising its potential. The goal of the present study is to answer the following questions: (i) How does temperature influence NH_4_^+^-N removal under substrate-limiting and non-limiting conditions in the MABR? (ii) Do temperature effects involve changes in the microbial community composition, and (iii) What is the role of mass transfer in the temperature dependence of nitrification in the MABR? This was achieved by operating a laboratory scale MABR system at various temperatures, monitoring its nitrification performance, performing microbial community analysis on biofilm samples, and mathematical modelling of the process.

## 2. Materials and methods

### 2.1. Experimental set-up

A laboratory scale MABR system comprising of two polycarbonate columns (BR1 and BR2), each with a membrane bundle of 540 hollow fibres made from polydimethylsiloxane (PDMS) was used for this study. Each of the fibres were 1.9 m in length with an internal diameter of approximately 280 μm and an external diameter of 550 μm, yielding a surface area of 1.87 m^2^. Oxygen was supplied to the bunches (from top to bottom) *via* a compressed air line and the pressure was controlled by a regulator (IMI Norgren Model No: 11400-2G-PC100 set ≈ 235 mbar). A variable area flow metre with integrated needle valve [Influx Measurements Ltd., UK, range (20–250 cm^3^/min)] was used on the inlet and outlet side of each bundle, where the inlet flow metre controlled the air flowrate at ≈ 210 cm^3^/min and the outlet flow metre had the bunches pressurised.

The total volume of the reactor was 28 L, the recirculation flow through the columns over the biofilm was set at 2 L/min (IFM electronic Model No. SM7100). A reservoir at the back of the reactor drained into two pumps that recirculated the bulk liquid through the two columns and back to the reservoir. The temperature of the reactor was controlled by a heating immersion circulator (Julabo Corio C, Julabo GmbH, Germany) placed in the reservoir which also had a stirrer that helped to mix the liquid in the reservoir. An overflow from the reservoir was discharged as effluent through the waste pipeline when the system was in continuous feed mode.

The system was operated in continuous mode for 4 weeks at 24°C, before carrying out the batch experiments. The batch experiments were carried out at 3–7 days intervals, with the exception of the first test at 8°C, which was performed 18 days after the preceding test. In between and after the batch experiments, continuous operation was maintained. The synthetic feed was prepared by dissolving NH_4_HCO_3_, Na_2_HPO_4_(H_2_O)_2_, and NaHCO_3_ in tap water.

### 2.2. Experimental design and analysis

To carry out batch tests, the continuous feed to the reactor was stopped 1 h prior to the tests. The reactor was rinsed with tap water, drained, and then connected to a tank with synthetic sewage (Table 1) prepared for the experiment. Liquid was recycled between the tank and the reactor’s reservoir using a submersible pump (Supplementary Figure 1). The system was allowed to stabilise for 10 min to attain the desired temperature (30, 24, 18, 12, and 8°C) and ensuring the additional volume from the columns (10.3 L) mixed with the batch feed solution. A chiller and heat exchanger (Model: Lowara BPX LBP410-060) were used to achieve temperatures of ≤24°C. A total of 20 ml samples were collected from the centre of the feed tank every 15 min.

**TABLE 1:** Constituents of the synthetic feed.

Constituent	Concentration (mg/L)
Ammonium bicarbonate (NH_4_HCO_3_)	284 (50 as N)
Disodium phosphate [Na_2_HPO_4_(H_2_O)_2_]	17 (3 as P)
Sodium bicarbonate (NaHCO_3_)	453

During the first run, the experiments were started at 30°C and the temperature was decreased to 8°C in successive steps (24, 18, and 12°C). Once the experiments at 8°C was complete, the temperature in the reactor was increased again to get duplicate results at each temperature (second run). The samples were centrifuged at 10,000 RPM for 15 min, then analysed for NO_2_^–^-N, NO_3_^–^-N, and NH_4_^+^-N concentrations with Hach LCK cuvette tests and spectrophotometer as described by Ravishankar et al. (2022).

### 2.3. Calculations

The ammonia removal rate was calculated by linear fitting to the concentration and time datapoints (Supplementary Figure 2) using the scaled Levenberg–Marquardt method in QtiPlot software, version 1.0.0.-rc13 (Ion Vasilief, Romania). The obtained rate of concentration change (g N m^–3^ d^–1^) was multiplied by the total liquid volume (m^3^) and divided by the membrane surface area (m^2^) to obtain an area specific removal rate (g N m^–2^ d^–1^). The rate was calculated for two concentration ranges: between 10 and 20 mg N/L and between 2.5 and 10 mg N/L. An average rate at each temperature was calculated using the results obtained from the first and second batch experiments. Temperature dependence was described by fitting an Arrhenius-type correlation to the data using QtiPlot:


$$R_{T_{1}}=R_{T_{0}}\,\theta^{\left(T_{1}-T_{0}\right)}$$

and by calculating the percent increase of reaction rate per °C of temperature increase:


$$100\times \frac{\left(R_{T_{1}}-R_{T_{0}}\right)/R_{T_{0}}}{T_{1}-T_{0}}$$

### 2.4. Mathematical modelling

A mathematical model of the MABR was compiled in Aquasim (Reichert, 1994). Growth of AOO and NOO in the one-dimensional biofilm was modelled using the rate equations and constants from Jones et al. (2007). The model was simplified by not including pH and alkalinity dependence. The biofilm was connected at its base to a mixed gas compartment by a diffusive link modelling the transport of oxygen across a PDMS membrane. The following temperature sensitive parameters were included in the model: Henry’s law constant of oxygen, diffusivity constants of soluble components, membrane permeability, maximum growth rate, and decay coefficient of AOO and NOO (the handling of the temperature dependence of these parameters is described in Supplementary material). Biofilm thickness was kept constant by specifying the surface detachment rate equal to the growth velocity of the biofilm.

Simulations were carried out at eight combinations of two parameters: biofilm thickness (50 and 150 μm), liquid boundary layer (LBL) thickness (50 and 250 μm), and temperature (8 and 30°C). The reaction rate was evaluated at 35 mg/L bulk liquid ammonium representing non-limiting conditions, as well as between 0.1 and 1.0 mg/L ammonium representing significant electron donor limitation. The influence of temperature for the various combinations of biofilm thickness and LBL thickness was calculated as % change of rate per °C change of temperature.

A sensitivity analysis was performed at the same eight combinations of parameters. The sensitivity of the model to the diffusivity constants of oxygen and ammonia was investigated. For each case, the diffusivity constant of either was changed by ±25%. The relative sensitivity was calculated for the ammonia removal rate at 35 mg/L ammonia concentration and the correlation of ammonia removal rate and ammonia concentration in the range of 0.1–1.0 mg/L.

### 2.5. Microbial community analysis

Membrane strands were obtained from the reactor (BR1) at 24 and 8°C. Samples at 24°C were obtained 2 weeks before starting the batch experiments, samples at 8°C were retrieved 4 weeks after the last batch experiment, following a gradual temperature decrease and operating at 8°C for 2 weeks. The biofilm attached on the membranes was dislodged using a vortex mixer, following which samples were centrifuged at 10,000 × *g* for 5 min. The pellet was used to extract the total genomic DNA using DNeasy PowerSoil Kit (Qiagen, Germany). To confirm the presence of DNA, the extracted DNA was visualised by UV excitation after running the samples in agarose gel electrophoresis (1%) with 1 mg L^–1^ GelRed™ (Bioscience) with Hyperladder IV (Bioline) as a molecular weight marker. The concentrated DNA was analysed using a Qubit™ 2.0 Fluorometer.

The obtained DNA was sent to Novogene Institute (UK) for 16S rRNA sequencing. 16S rRNA genes of distinct regions were amplified using specific primers (e.g., 16S V4: 515F-806R) using Phusion^®^ High-Fidelity PCR Master Mix (New England Biolabs) (Jiang et al., 2019). The amplified PCR products were run on 2% agarose gel electrophoresis and samples with 400–450 base pairs were chosen for purification and library generation using NEBNext^®^ UltraTM DNA Library Prep Kit for Illumina. Later the library was quantified *via* Qubit and Q-PCR, was analysed by Illumina platform.

The sequence data processing and filtering of raw tags was done as described in detail by Jiang et al. (2019). The tags were compared to the reference SILVA database^1^ using UCHIME algorithm to detect the chimaera sequences (Edgar et al., 2011). The effective tags were then obtained by removing the chimaera sequences.

Uparse software was used to analyse the effective tags for sequence analysis (Edgar, 2013). Sequences with ≥97% similarity were assigned to the same Operational Taxonomic Units (OTUs). Representative sequence for each OTU was annotated using Qiime (Version 1.7.0) in Mothur method against the SSUrRNA database of SILVA database for species annotation at each taxonomic rank (kingdom, phylum, class, order, family, genus, and species) (Altschul et al., 1990; Wang et al., 2007; Quast et al., 2012). To obtain the phylogenetic relationship of all OTUs representative sequences, multiple sequence alignment was conducted using MUSCLE (version 3.8.31) software. OTUs abundance information was normalised as described in Jiang et al. (2019). Subsequent analysis (alpha and beta diversity) was performed based on the normalised data. All the indices in the samples of alpha diversity were calculated with QIIME (version 1.7.0) and displayed with R software (version 2.15.3). Principal component analysis was adopted to show the beta diversity using the FactoMineR package and ggplot2 package in R software (version 2.15.3).

Metagenomes were predicted from the obtained OTU using PICRUSt. The OTU generated by the Mothur method was formatted as BIOM files as input for PICRUSt. The OTUs abundance mapped to SILVA database was used as input by PICRUSt for metagenomes prediction (Mukherjee et al., 2017). Marker gene data was compared with the reference genomes in database Kyoto Encyclopaedia of Genes and Genomes (KEGG) to predict the metabolic pathway (Yin and Wang, 2021).

## 3. Results

### 3.1. Ammonium removal rate

**FIGURE 1:**
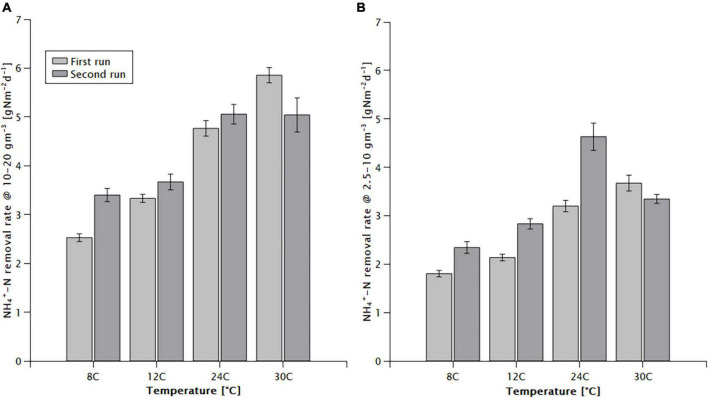
Effect of temperature on the NH_4_^+^-N removal rate of the MABR in the **(A)** high (10–20 mg/L) and **(B)** low (2.5–10 mg/L) initial NH_4_^+^ -N concentration ranges.

The average area specific NH_4_^+^-N removal rate in the 10–20 mg/L concentration range was 2.96–5.45 g N m^–2^ d^–1^ (Figure 1A), and for 2.5–10 mg/L it was 2.07–3.91 g N m^–2^ d^–1^ (Figure 1B), showing a positive correlation with the temperature (Figure 1). When expressed with an Arrhenius-type relationship, coefficients (θ) of 1.026 (±0.003) and 1.024 (±0.010) were obtained, respectively. The low value of the Arrhenius coefficient indicates a low temperature dependence, the relationship does not follow the Arrhenius-type relationship precisely, underestimating the effect of temperature at lower, and overestimating it at higher temperatures. For example, in the 10–20 mg/L concentration range, between 24 and 30°C the increase of the maximum removal rate was only 1.4% per °C, while between 12 and 24°C it was 3.4% per °C and between 8 and 12°C it amounted to 4.5% per °C.

High nitrification rates were achieved at a low temperature (Figure 1). However, the bulk liquid ammonium concentration remained an important factor influencing the rate. The rate at a low temperature (8°C) and high concentration (10–20 mg/L) was measured at 2.96 g Nm^–2^ d^–1^, decreasing to 2.07 g Nm^–2^ d^–1^ at the lower concentrations (2.5–10 mg/L).

**FIGURE 2:**
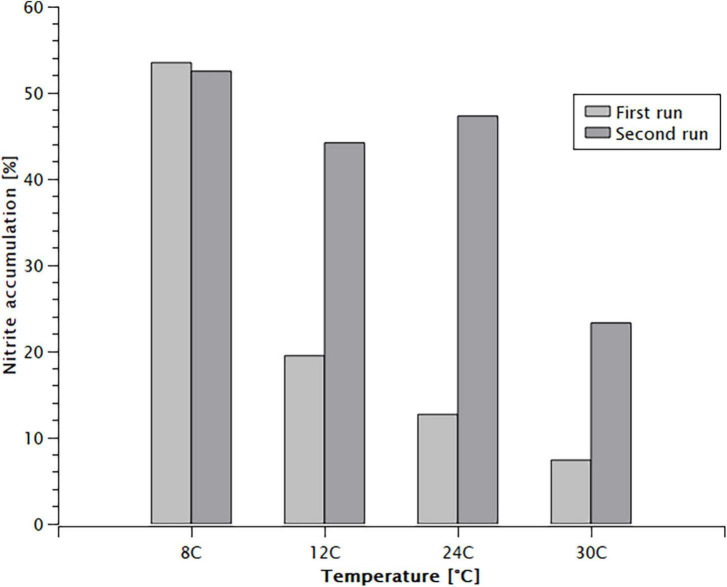
Nitrite accumulation by the end of the batch experiments.

Nitrite accumulation was observed in all batch tests (Figure 2). The fraction of nitrite in the oxidised nitrogen at the end of the tests was inversely proportional with the temperature. During the first run of experiments the accumulation of nitrite increased from 7.4% at 30°C to 53.5% at 8°C. Once the temperature was increased back, the accumulation dropped gradually to 23.3% at 30°C. It took 5 weeks of continuous operation at 24°C for the nitrite accumulation to return to the 10 and 15% range achieved before the batch tests were performed.

### 3.2. Bacterial community composition

**FIGURE 3:**
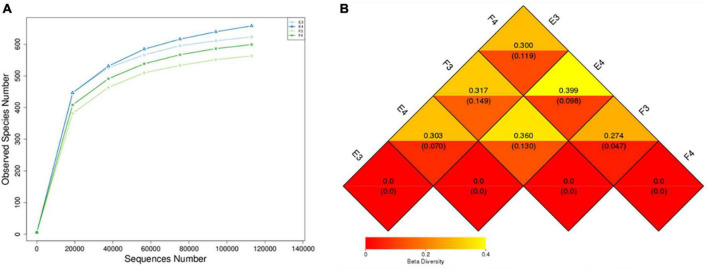
**(A)** Alpha biodiversity indicating species rarefaction curve and **(B)** beta biodiversity heat map (upper and lower values represent the unweighted and weighted Unifrac distance) of biomass samples from the MABR. E3 and E4 are duplicate samples taken at 24°C, F3 and F4 are duplicate samples taken at 8°C.

Microbial community composition at high (24°C) and low (8°C) temperatures were analysed on the Illumina platform. Table 2 shows the alpha diversity index of the samples. E3 and E4 are duplicate samples at 24°C and F3 and F4 correspond to duplicate samples at 8°C. Chao 1 and ACE were higher for samples at 24°C compared to 8°C, indicating a richer community at higher temperature. Higher Shannon and Simpson indices at 8°C compared to 24°C represented a more homogeneous community at the lower temperature. The alpha biodiversity species rarefaction curves (Figure 3A) illustrate the same observation with community richness observed for samples E3 and E4 (24°C) against F3 and F4 (8°C).

**TABLE 2:** Alpha diversity analysis index at 97% clustering threshold.

Parameter	Samples	
	**24°C – E3 and E4** **(averaged)**	**8°C – F3 and F4** **(averaged)**
Operational taxonomical units (OTU) numbers	658	609
Shannon	4.092	4.202
Simpson	0.736	0.787
Chao1 richness	709.54	613.25
ACE richness	687.72	615.27

The weighted Unifrac distance between samples from different temperatures was between 0.098 and 0.149, while it was 0.047 and 0.070 between samples from the same temperature (Figure 3B). This showed a difference in microbial community composition between samples taken from the 24 and 8°C batch tests.

**FIGURE 4:**
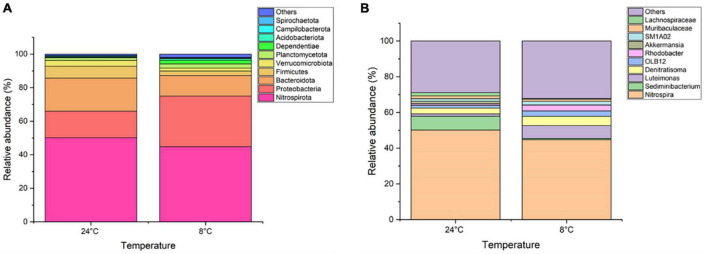
Relative abundance of top 10 taxa **(A)** phyla level and **(B)** genera level of biomass samples taken from the MABR at 8 and 24°C.

Figure 4A shows the top 10 phyla found across all four samples within the bacterial community. Of the nine phyla, *Nitrospirota* was predominant followed by *Proteobacteria* and *Bacteroidota*, with an overall contribution of >85% in all the samples. The proportion of *Nitrospirota* (also known as *Nitrospirae*) decreased by 6% when the MABR operational temperature was decreased from 24 to 8°C.

Within the genus level, almost 239 microbial genera were detected at both temperatures. An average of 12 and 15% of the samples were unclassified for 24 and 8°C, respectively. The top 10 genera for both temperatures are presented in Figure 4B. *Nitrospira* was predominant in both samples, while *Sediminibacterium* was prevalent in the 24°C samples (at 7.5%). The *Nitrosomonas* genus representing the ammonia oxidising proteobacteria was present at 0.8 and 0.4% at 24 and 8°C, respectively.

Further species level analysis illustrated the existence of the top four species *Nitrospira defluvii, Akkermansia muciniphila, Nitrosomonas oligotropha*, and *Pseudoxanthomonas mexicana*. Nitrite oxidising bacterium (NOB) *N. defluvii* was found in abundance in both samples (>45%). Ammonia oxidising bacteria (AOB) *N. oligotropha* abundance was at 0.8 and 0.3% observed in samples obtained at 24 and 8°C. The overall abundance of AOBs and NOBs were reported to be less than 0.25 and 0.35%, respectively.

### 3.3. Predictive functional analysis

The relative abundance of genes didn’t vary considerably between the two temperatures investigated. The metabolism related genes were in the highest abundance (∼50%) followed by genetic information processing (∼17%) and environmental information processing (∼11%) in both samples tested. Looking specifically at the abundance of nitrogen metabolism related genes, a 0.9% of relative abundance was noted in both samples at 24 and 8°C.

**FIGURE 5:**
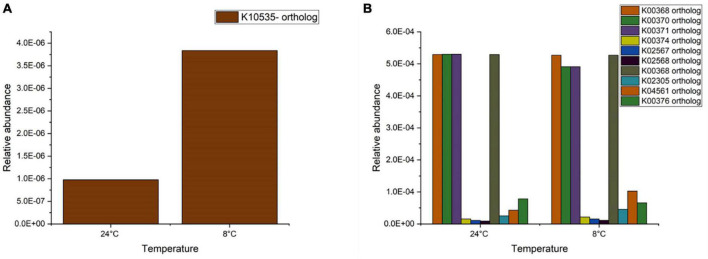
Relative abundance of **(A)** nitrification and **(B)** denitrification orthologues present in biomass samples at 24 and 8°C.

Further analysis on nitrogen metabolism was conducted through comparing the functional orthologues with the KEGG database. For nitrification, the functional orthologues that are reported in conversion of ammonia to hydroxylamine are K10944, K10945, and K10946, corresponding to the genes *pmoA-amoA, pmoB-amoB*, and *pmoC-amoC* (representing ammonia monooxygenase). Further, hydroxylamine is converted to nitrite by hydroxylamine dehydrogenase represented by the orthologue K10535, corresponding to the gene *hao*. Oxidation of nitrite to nitrate is carried out by nitrite oxidoreductase (nxrA and nxrB), represented by K00370 and K00371. The orthologues K10944, K10945, and K10946 were not identified in the samples (24 and 8°C) examined. The orthologue K10535 (gene *hao*) was noted to be present in both samples and its relative abundance was threefold higher at 8°C than at 24°C (Figure 5A). This high relative abundance meant a higher activity of the hydroxylamine dehydrogenase and conversion of hydroxylamine to nitrite. The abundance of the orthologues K00370 and K00371 remained similar for both temperatures. Although COD was not supplied in the present study and no denitrification was observed, denitrification related functional KEGG orthologues were noted to be present in all samples (Figure 5B). The abundance of orthologue K00368 (nitrite reductase-*nirK*) did not change with temperature. The periplasmic nitrate reductase (K02567-*napA*) and cytochrome c-type protein involved in nitrate reduction (K02568-*napB*) showed an increase at 8°C compared to 24°C. The relative abundance of nitric oxide reductase (K02305-*norC* and K04561-*norB*) was noted to increase around two to sixfold at 8°C. The orthologue K00376 (nitrous oxide-*nosZ*) was noted to decrease when the temperature decreased from 24 to 8°C. It should be noted that these PICRUSt derived predictive results on gene abundance need to be further validated based on actual gene expression using transcriptomics.

### 3.4. Modelling

**FIGURE 6:**
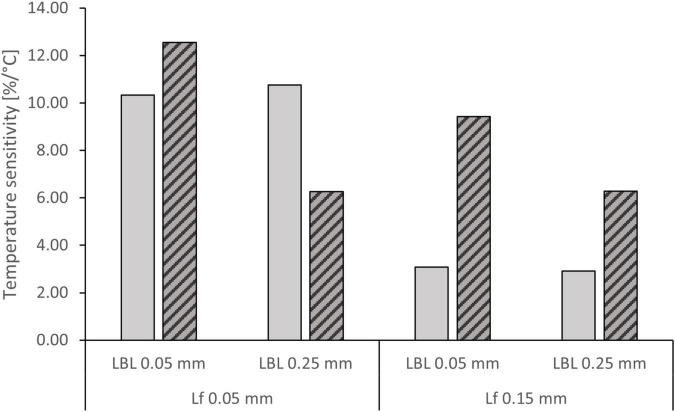
Temperature sensitivity of NH_4_^+^-N removal rate at 35 mg/L (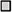) and 0.1–1.0 mg/L (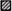) NH_4_^+^-N concentration at various combinations of biofilm thickness (Lf) and boundary layer thickness (LBL).

The simulations predicted maximum nitrification rates of 1.76–2.80 g N m^–2^ d^–1^ at 8°C and 4.49–5.95 g N m^–2^ d^–1^ at 30°C (Table 3). Both biofilm thickness and the LBL thickness were found to influence temperature dependence (Figure 6). When ammonium was non-limiting (35 mg/L), low biofilm thickness resulted in a higher temperature dependence of 10.5% per °C compared to 3% per °C, and the thickness of the boundary layer did not influence temperature sensitivity significantly. When ammonium was limiting (0.1–1.0 mg/L), the biofilm thickness had a smaller influence on temperature dependence, but showed a similar trend of higher sensitivity at lower biofilm thickness. At the same time, the LBL thickness had an increased impact on temperature dependence compared to non-limiting concentrations (Figure 6).

**TABLE 3:** Model predicted nitrification rates under various combinations of temperature (T), biofilm thickness (Lf), and liquid boundary layer thickness (LBL).

T (°C)	8	30	8	30	8	30	8	30
Lf (m)	0.00005	0.00005	0.00015	0.00015	0.00005	0.00005	0.00015	0.00015
LBL (m)	0.00005	0.00005	0.00005	0.00005	0.00025	0.00025	0.00025	0.00025
Rate at 35 mg/L (g N m^–2^ d^–1^)	1.82	5.95	2.80	4.69	1.76	5.91	2.74	4.49

### 3.5. Sensitivity analysis

**FIGURE 7:**
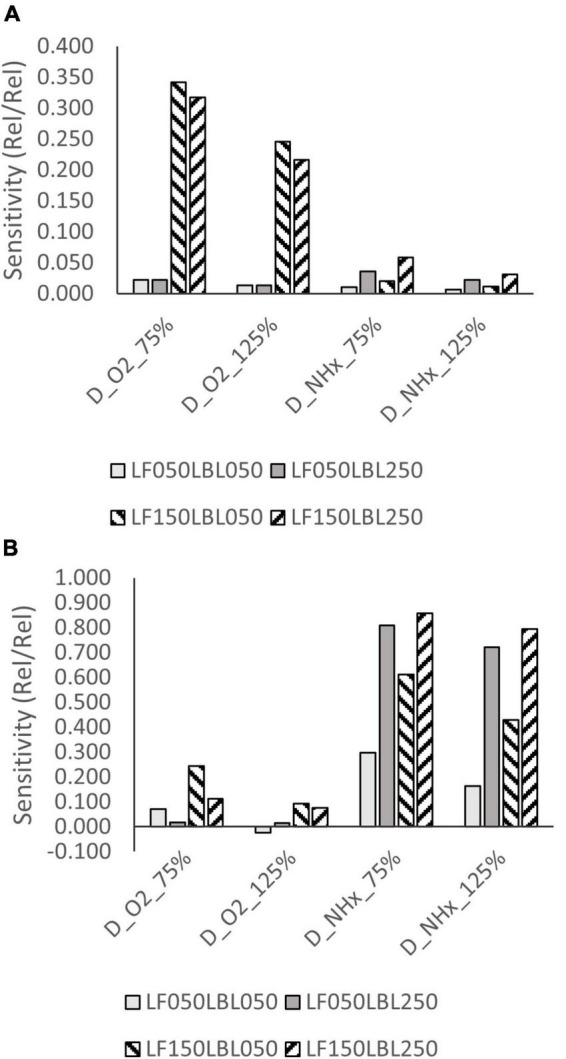
Sensitivity of NH_4_^+^-N removal rate to the diffusivity constant of oxygen and ammonium at **(A)** 8°C and 35 mg/L, and **(B)** at 8°C and 0.1-1.0 mg/L NH_4_^+^-N concentration.

Moderately high sensitivity (>0.200) to oxygen diffusivity was observed at high ammonium concentrations and high biofilm thickness at 8°C (Figure 7A). Sensitivity at high temperature and high ammonium concentration was low (<0.200). Sensitivity to the diffusivity of ammonium was high (>0.600) at low ammonium concentration and high LBL thickness, both at 8 and 30°C (Figure 7B).

## 4. Discussion

### 4.1. Low temperature nitrification

The nitrification rates achieved in this study (1.8–5.8 g N m^–2^ d^–1^) can be considered characteristic of biofilm systems operated at low organic loads and compare well to the literature values introduced earlier. These high reported rates have promoted the use of MABRs to augment the ammonia removal performance of existing suspended growth processes. For example, Houweling et al. (2017) reported area specific nitrification rates of 1.7–2.3 g N m^–2^ d^–1^ in pilot and full-scale hybrid MABR/AS systems at moderate temperatures (15–20°C). Results from this study indicate that at low temperatures the MABR can achieve a nitrification performance similar or better compared to other biofilm processes. For comparison, Ha et al. (2010) obtained a maximum rate of 0.35–0.50 kg N m^–3^ d^–1^ (0.38–0.54 g N m^–2^ d^–1^) in biological aerated filters at 6.5 and 13°C, at a specific surface area of 930 m^2^ m^–3^ and a COD:N ratio of 2. Several studies investigated the performance of biofilms at extremely low temperatures as well, and while a kinetic threshold was found at around 4°C, nitrification could be maintained at as low as 1°C (Delatolla et al., 2009; Hoang et al., 2014; Young et al., 2017; Ahmed and Delatolla, 2021).

At higher temperatures the counter-diffusional, direct oxygen delivery of the MABR enables high oxygen transfer rates despite the reduced oxygen solubility in the water, without imposing prohibitive energy costs on the process. A significantly higher nitrification rate can be reached this way, giving an advantage over co-diffusional biofilms (He et al., 2021). Upgrading wastewater treatment plants with a strong seasonal variation of both load and wastewater temperature in particular may benefit from the application of MABR technology.

### 4.2. Temperature influence on biofilms

The temperature dependence observed in this study was low compared to that of the growth rate of ammonium oxidisers. Similar values could be observed in other biofilm processes however, it was noted that the duration of exposure to low temperature can influence the temperature dependence (Delatolla et al., 2009; Ashkanani et al., 2019). In certain cases, no temperature dependence of nitrification was observed in biofilm processes. Long et al. (2020) used full-scale and pilot scale MABR data to model the seasonal changes in nitrification performance and could not detect a correlation with temperature. However, they acknowledged that changes in the biofilm may have masked its effect. Such changes could potentially involve the structure (thickness, density, and morphology) or the community composition of the biofilm, affecting the mass transfer characteristics and the inherent reaction kinetics as well.

In the present experimental work, no significant difference could be found between temperature dependence of the nitrification rate at low and high ammonium concentrations, the low θ values (1.024 and 1.026) indicate mass transfer limitations in both cases. The observed deviation from the Arrhenius correlation can also be attributed to the mass transfer limitation. Jang and Byun (2013) reported a similar trend of the temperature impact being more significant at lower temperatures than at higher ones. The modelling work showed that increasing the external mass transfer resistance (LBL thickness) results in a reduced temperature dependence in case of ammonium limitation, but not when ammonium is non-limiting. Increased internal mass transfer resistance (biofilm thickness) on the other hand affects the nitrification rate more significantly when ammonium is non-limiting, and oxygen availability becomes the rate limiting factor (Supplementary Figure 3). The biofilm thickness affected the nitrification rate *via* the amount of active nitrifying biomass as well, which became a limiting factor under low temperature and high ammonium concentration. At high temperatures the ammonium mass transfer limitation at low ammonium concentrations is compensated by the increased surface area of a thicker cylindrical biofilm (Supplementary Figure 4). There is an inverse correlation between temperature sensitivity of nitrification and its sensitivity to the diffusivity of oxygen and ammonia as well, indicating that mass transfer resistance is responsible for the reduced impact of temperature.

The influence of mass transfer on the temperature dependence in biofilm processes was suggested by multiple studies. For example, when treating a secondary effluent with MBBRs, Salvetti et al. (2006) found that temperature had no impact on the nitrification rate when ammonium was the limiting substrate, but under oxygen limiting conditions the temperature dependence was apparent, resulting in an average coefficient (θ) of 1.058. Interestingly, the opposite was observed by Zhu and Chen (2002) in a biofilter with higher temperature dependence in case of ammonium limitation. Reinforcing our observations, the temperature dependence of nitrification was found to be similar to its impact on diffusion rather than on the biological reaction by Hem et al. (1994). Ashkanani et al. (2019) reported that carriers with a high specific surface area, which are prone to clogging with biomass, showing less dependence of the nitrification rate on temperature than carriers with lower specific surface area but no clogging. These examples all support the theoretical considerations voiced by Characklis and Gujer (1979) about the influence of mass transfer phenomena on temperature dependence of biofilm kinetics.

We have observed a difference between the first and second run of experiments (Figure 1), which may reflect changes in the mass transfer characteristics of the biofilm. While no measurements were carried out, the biofilm thickness could potentially increase over time, as no scouring was applied to minimise disturbances to the system. An increased biofilm thickness could explain the reduced temperature dependence of the nitrification rate in the second run, with higher minimum and lower maximum values achieved (Figure 6). At the same time changes in the activity and composition of the microbial community (Figure 5) may have contributed to the observed difference as well. The community analysis suggested an increase in the ammonium oxidising activity following the reduction of temperature to 8°C, despite the reduction of the relative abundance of *Nitrosomonas*. This, together with the reduced diversity indicated a moderate level of community shift taking place.

### 4.3. Nitrifier community in the MABR

The phylum level composition of the biofilm was quite consistent with the literature as these are the common phyla that are present in nitrifying wastewater treatment plants across the world. Chemolithoautotrophic AOBs of the β-subgroup of *Proteobacteria* have been well reported as a major nitrifying community in different ecosystems (Kowalchuk et al., 2000; Laanbroek, 2012; Young et al., 2017). The *Bacteroidota* phylum prevailing with heterotrophic bacterial groups have been confirmed in different aqueous environments (Díez-Vives et al., 2012; Poghosyan et al., 2020) and could degrade particulate organic matter present in water bodies (Dorador et al., 2009). The *nirK* genes have been reported to be involved in both denitrification and ammonia oxidation (Cantera and Stein, 2007; Kozlowski et al., 2014; Long et al., 2015), so the abundance of the K00368 orthologue is inconclusive with regards to its significance in either process. The low abundance of ammonium oxidisers shown in our samples has been observed previously in activated sludge wastewater treatment plants. Findings of the present study correspond to *Nitrosomonas* sp. and *Nitrospira* sp. being the most abundant nitrifying bacteria in activated sludge (Kruglova et al., 2020) in Nordic regions.

The increased nitrite accumulation at the lower temperatures (Figure 2) indicates that the activity of NOB was more affected by cold temperature than that of AOB. In contrast, Knowles et al. (1965) found that nitrite oxidation was less impacted by temperature than ammonia oxidation, however their findings were specific to *Nitrobacter*. The dominant NOB was *N. defluvii* in this case, which may be more sensitive to the temperature changes experienced in this experiment. Their abundance dropped by 6%, which correlates with their reduced activity. A similar change in *Nitrospira* abundance was presented by Young et al. (2017) when reducing the operating temperature from 20 to 1°C. The accumulation of nitrite was not present in the simulated results, adjustment of NOB growth in the kinetic model would be necessary to capture the behaviour observed in the experiments.

## 5. Conclusion

Nitrification at various temperatures (8–30°C) in an MABR was investigated by lab scale batch tests, microbial community analysis and mathematical modelling. The main objectives were to investigate the impact of temperature on ammonium oxidation rate and the microbial community, and the role of mass transfer in the temperature dependence of nitrification in counter-diffusional biofilms.

The nitrification performance of the membrane-aerated biofilm at low temperature is comparable or even favourable to other biofilm processes. A volumetric ammonia removal rate of 1.07 kg N m^–3^ d^–1^ and an area specific rate of 3.08 g N m^–2^ d^–1^ were achieved at 8°C temperature and 10–20 g m^–3^ effluent NH_4_^+^-N concentration. The observed temperature dependence of the reaction was lower than that reported for suspended cultures and activated sludge, with a correlation factor (θ) of 1.024–1.026. The correlation deviated from the Arrhenius-type relationship, as a stronger influence was observed at the lower end of the investigated temperature range. The microbial community was dominated by *N. defluvii*. However, nitrite oxidation activity was reduced at lower temperatures and nitrite accumulation was observed. The relative abundance of confirmed ammonia oxidisers was low, but an increase in the relative abundance of the orthologue K10535 (gene *hao*) from 24 to 8°C was detected. The diversity of the community decreased when going from 24 to 8°C. The temperature sensitivity of nitrification is inversely proportional to the influence of mass transfer of both oxygen and ammonia. Mass transfer resistance is largely responsible for the observed low temperature sensitivity. High biofilm thickness can result in more significant mass transfer limitations, leading to lower temperature sensitivity, but the increasing amount of biomass and the larger biofilm surface area in a cylindrical biofilm geometry both influence the relationship under biomass and ammonium limited conditions, respectively.

## Data availability statement

The sequencing data presented in this study are deposited in the NCBI repository, accession number PRJNA954668.

## Author contributions

JA designed and operated the experimental system, set up and carried out the experiments, and contributed to writing the Materials and methods section. HR assisted with the reactor operations, led the microbial community analysis, and contributed to writing of the Materials and methods, Results, and Discussion sections. AN led data interrogation, performed the mathematical modelling, and contributed to writing all sections. PL contributed to data analysis, proofreading, corrections to all sections, and funding acquisition. BH contributed to data analysis, proofreading, and corrections to all sections. All authors contributed to the article and approved the submitted version.

## Footnotes

1
http://www.arb-silva.de/
