# Supplementary material for: Temperature dependence of nitrification in a membrane-aerated biofilm reactor
Source: Front Microbiol. 2023 Apr 24;14:1114647. doi: 10.3389/fmicb.2023.1114647 (PMC10165249; doi:10.3389/fmicb.2023.1114647)

# Supplementary Information

## Experimental Setup

Supplementary Figure 1. Flow chart of the experimental setup during the batch experiments. When in continuous operation, the external reservoir was disconnected, and the synthetic feed was pumped into the mixing recirculation line in front of the impeller pump.

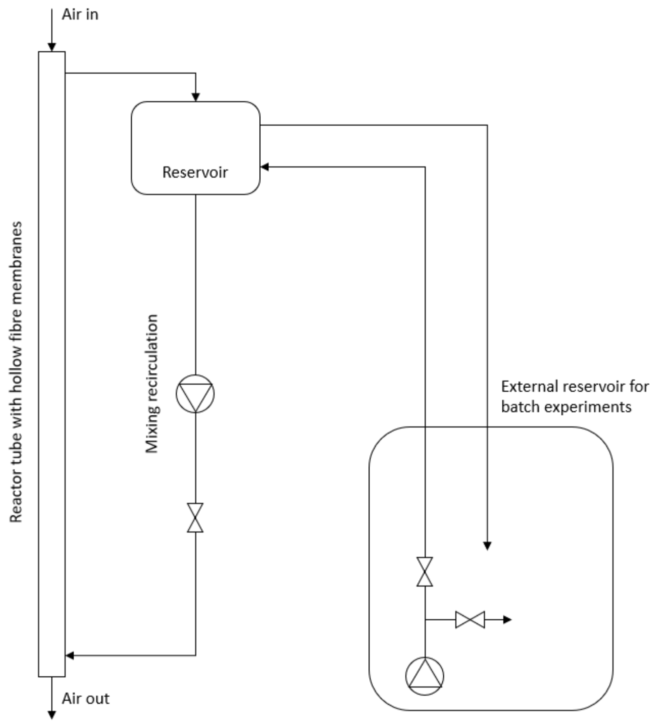


## Temperature Dependence of Model Parameters in the Mathematical Model of the MABR

Aerobic growth, aerobic and anoxic decay of ammonium oxidising and nitrite oxidising organisms was considered in the mathematical model of the biofilm. The maximum specific growth rate of both AOO and NOO depended on the temperature. The temperature dependent maximum specific growth rate was calculated from the rate at 20°C and the temperature dependence coefficient according to the following equation:

$$\mu_{AOO}^{T} = \mu_{AOO}^{20} \theta_{\mu,AOO}{}^{(T-20)}$$

The anaerobic and anoxic decay rates were handled in an identical fashion:

$$b_{AOO,O2}^{T} = b_{AOO,O2}^{20} \theta_{b,AOO,O2}{}^{(T-20)}$$

Temperature dependence of the half-saturation constants was not considered.

(Jones, R.M., Dold, P.L., Takács, I., Chapman, K., Wett, B., Murthy, S., Shaughnessy, M.O., 2007. SIMULATION FOR OPERATION AND CONTROL OF REJECT WATER TREATMENT PROCESSES.

Proceedings of the Water Environment Federation 2007, 4357–4372. https://doi.org/10.2175/193864707787974599)

The mass transfer of the dissolved constituents in the biofilm and the liquid boundary layer was also affected by temperature. The diffusivity constants of the substrates were influenced by the temperature according to the following relationship:

$$D_S^T = D_S^{20} 2.71828^{-0.029(T-20)}$$

The transfer of oxygen gas across the membrane was temperature dependent as well. Both the membranes permeability and oxygen's solubility were affected.

Oxygen permeability in PDMS was calculated according to:

$$P = P_0 e^{\left(-\frac{E_P}{RT}\right)}$$

where $P_0$ is the pre-exponential factor of permeation, $E_P$ is the activation energy for permeation, and R is the gas constant (Komatsuka, T., Nagai, K. Temperature Dependence on Gas Permeability and Permselectivity of Poly(lactic acid) Blend Membranes. Polym J 41, 455–458 (2009). https://doi.org/10.1295/polymj.PJ2008266).

The temperature dependence of oxygen's Henry's law constant was calculated as:

$$k_H^T = k_H^\theta e^{\left[\frac{-\Delta_{soln}H}{R}\left(\frac{1}{T}-\frac{1}{T^\theta}\right)\right]}$$

where $T^\theta$ is the standard temperature (298.15 K) and $\Delta_{soln}H$ is the enthalpy of solution (R. Sander, Compilation of Henry's Law Constants for Inorganic and Organic Species of Potential Importance in Environmental Chemistry, version 3, doi:10.5194/acp-15-4399-2015).

## Batch Test Results

Supplementary Figure 2. Summary of the change of ammonium concentration during the batch experiments.

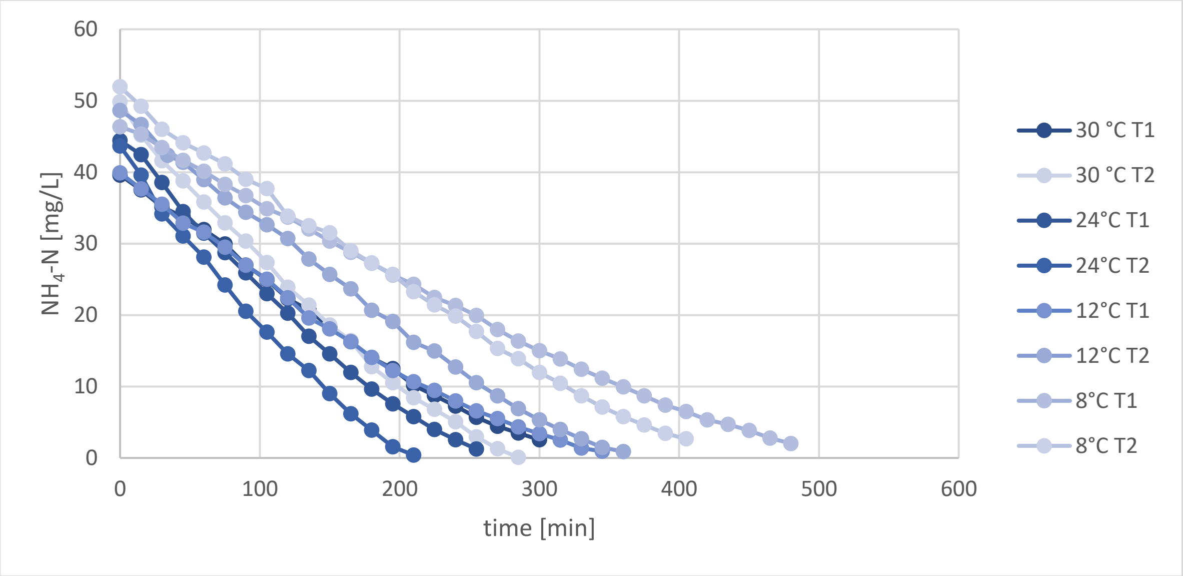

## Modelling Results of Temperature and Mass Transfer Resistance Effects

Supplementary Figure 3. Effect of changing the diffusion coefficient of $O_2$ or $NH_x$ on the kinetics of ammonium removal at 0.150 mm biofilm thickness, 0.050 mm boundary layer thickness and 8°C temperature.

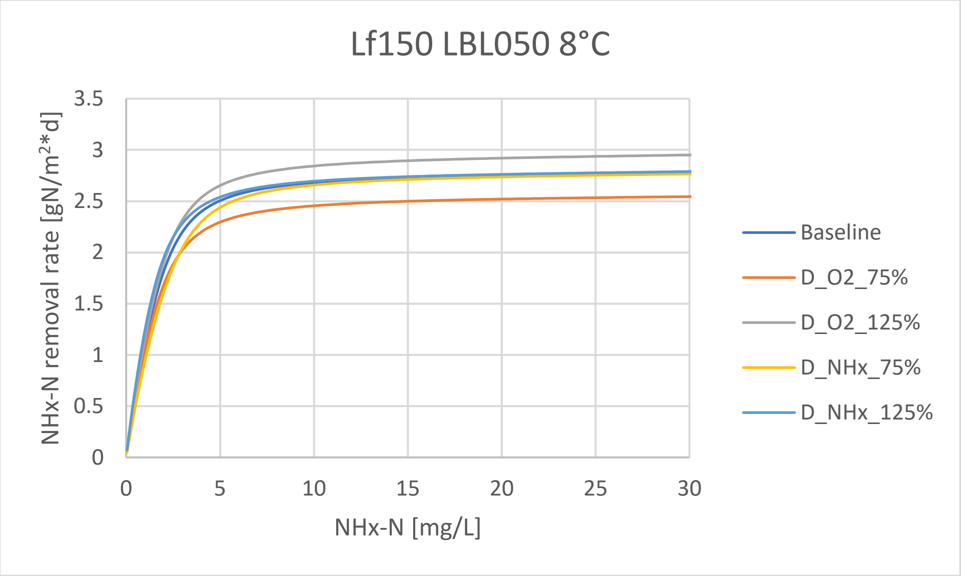


Supplementary Figure 4. Effect of the increased biofilm surface area with a cylindrical geometry at high biofilm thickness and low ammonium concentration.

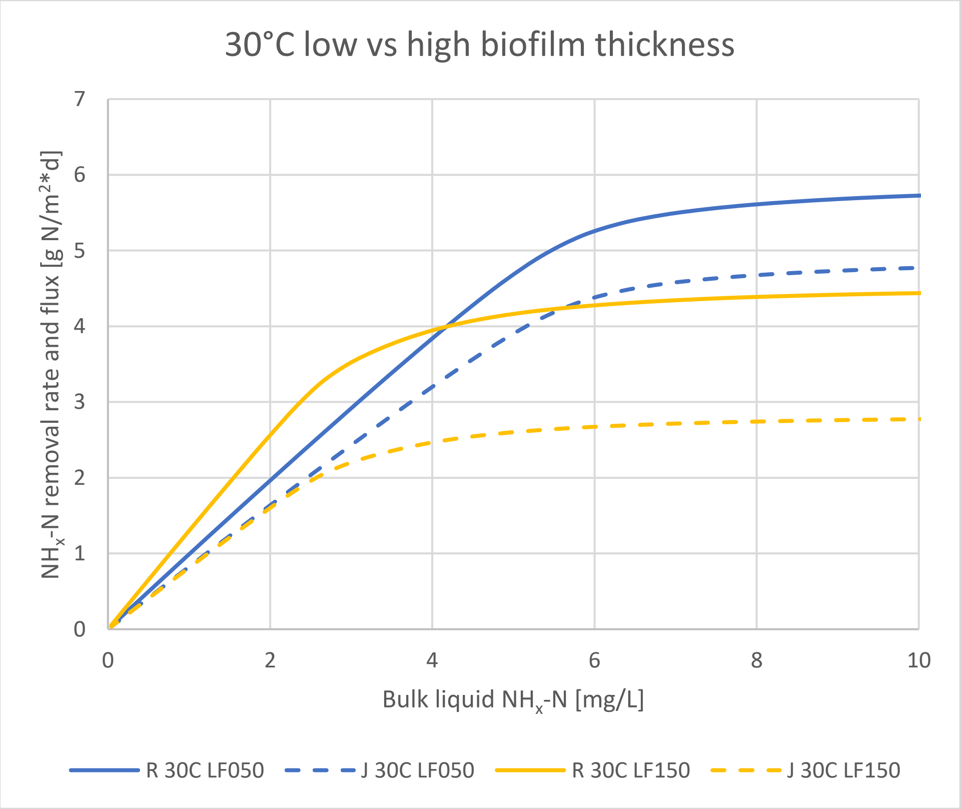

Supplement: Supplementary file 1 [file Data_Sheet_1.pdf]
